# Brain response to luminance-based and motion-based stimulation using inter-modulation frequencies

**DOI:** 10.1371/journal.pone.0188073

**Published:** 2017-11-15

**Authors:** Xin Zhang, Guanghua Xu, Jun Xie, Xun Zhang

**Affiliations:** 1 School of Mechanical Engineering, Xi’an Jiaotong University, Xi’an, China; 2 State Key Laboratory for Manufacturing Systems Engineering, Xi'an Jiaotong University, Xi'an, China; Chinese Academy of Sciences, CHINA

## Abstract

Steady state visual evoked potential (SSVEP)-based brain computer interface (BCI) has advantages of high information transfer rate (ITR), less electrodes and little training. So it has been widely investigated. However, the available stimulus frequencies are limited by brain responses. Simultaneous modulation of stimulus luminance is a novel method to resolve this problem. In this study, three experiments were devised to gain a deeper understanding of the brain response to the stimulation using inter-modulation frequencies. First, luminance-based stimulation using one to five inter-modulation frequencies was analyzed for the first time. The characteristics of the brain responses to the proposed stimulation were reported. Second, the motion-based stimulation with equal luminance using inter-modulation frequencies was also proposed for the first time. The response of the brain under these conditions were similar to that of luminance-based stimulation which can induce combination frequencies. And an elementary analysis was conducted to explain the reason of the occurrence of combination frequencies. Finally, the online test demonstrated the efficacy of our proposed two stimulation methods for BCI. The average ITRs reached 34.7836 bits/min and 39.2856 bits/min for luminance-based and motion-based stimulation respectively. This study demonstrated that the simultaneous modulation of stimulus luminance could extend to at least five frequencies to induce SSVEP and the brain response to the stimulus still maintained a certain positive correlation with luminance. And not only luminance-based stimulation, but also motion-based stimulation with equal luminance can elicit inter-modulation frequencies to effectively increase the number of targets for multi-class SSVEP.

## Introduction

Steady-state visual evoked potentials (SSVEP) are induced by visual stimulation containing a constant flickering frequency [1] ranging from 1 to 100 Hz [2]. The low frequency bands (5–12 Hz), middle frequency bands(12–30 Hz) and high frequency bands (30–60 Hz) are the three main frequency ranges. In the low and medium frequency bands, SSVEPs at 15 Hz show the largest amplitude [3]. Hence many SSVEP brain computer interface (BCI) systems select frequencies in the low and middle frequency bands as visual stimulation frequencies [4, 5]. However, visual stimulation at low frequencies can induce seizures and easily to cause eye discomfort. Furthermore, the low frequency bands overlap with EEG in the alpha band which may increase the difficulty of identification. Hence several research teams use frequencies in the high frequency bands as visual stimulation frequencies in SSVEP-BCI systems to avoid the risk of seizures and slow visual fatigue [6, 7]. However, accuracy and information transfer rate (ITR) are significantly lower due to lower amplitudes induced by high frequency stimulus [7]. What's more, limited by the screen refresh rate, cathode ray tube (CRT) and liquid crystal display (LCD) monitors can only produce limited available frequencies. These frequencies must be divided exactly by the screen refresh rate. What's more, single frequency stimulus may induce harmonic frequencies in SSVEP. That is to say, the fundamental frequency and the harmonic frequencies cannot be the stimulus frequency at the same time. To sum up, the effective visual stimulation frequencies are limited in SSVEP-BICs.

Recently, several studies have attempted to increase the number of targets by using limited frequencies. One method uses the phase information as using different phase lags can make different targets even using the same frequencies [8]. However, the number of phase coded targets is still limited by the refresh rate. Another method is coding multiple different frequencies into one target [9–12]. There are two ways to realize this coding method: sequential multiple frequencies flickering method and simultaneous multiple frequencies protocol. As to sequential multiple frequencies flickering method, the length of the stimulation time will increase as the number of frequencies is increased. Due to the shortages mentioned above, the simultaneous multiple frequencies protocol is proposed. Most studies use an LCD monitor or LEDs to present visual stimulation that combines two frequencies. However, in those studies, two LEDs are used to produce the dual frequency visual stimulation so that participants are required to focus their eyes on the middle of the two flickering points [11]. Otherwise, a shift of attention may be occur which may cause some frequency-peaks to be lost. Moreover, most studies have reported the characteristics of EEG response to the dual inter-modulation frequency visual stimulation. To gain a comprehensive understanding of luminance-based inter-modulation, the characteristics of EEG response to multiple inter-modulation frequencies visual stimulus is explored in this study.

SSVEPs mainly occur in the primary visual cortex from which there are two pathways to output information: ventral pathway and dorsal pathway [13]. The ventral pathway, also known as the P-pathway, is a color-sense-associated and object recognition pathway, which detects luminance and color. The dorsal pathway, also known as the M-pathway, is associated with spatial processing and motion recognition, and detects motion speed and direction [14, 15]. The existing studies on simultaneous multiple frequencies protocol are mostly based on changes of luminance and color [16] which induce potentials in the ventral pathway. The characteristics of steady-state motion visual evoked potential (SSMVEP) [17] in the dorsal pathway using simultaneous multiple frequencies protocol is still unknown.

In this study, two novel stimulation methods using inter-modulation frequencies are proposed. The first method is simultaneous modulation of stimulus luminance which can produce multiple inter-modulation frequencies using a single LED. Some interesting discoveries are shown in this study towards a comprehensive understanding of luminance-based inter-modulation. The other method is simultaneous modulation of stimulus motion which maintains equal luminance. The difference of the characteristics in the brain response between these two methods is discussed. Besides, both the methods mix multiple frequencies into one single target thus participants no longer need to shift their attention. Furthermore, an online test is implemented to demonstrate the feasibility of these two methods for SSVEP-BCIs. The results show that the brain response to stimulation using multiple inter-modulation frequencies still maintains a certain positive correlation with luminance and that both proposed methods can encode more target stimuli with limited frequencies.

## Materials and methods

### Ethics statement

Subjects were studied after giving informed written consent in accordance with a protocol approved by the institutional review board of Xi’an Jiaotong University.

### Design of luminance-based stimulation

In this study, a green straw hat LED is chosen as the visual stimulation. To avoid eye discomfort caused by the bright light from the middle of the LED, a hemispherical lampshade made of light diffusing material with 85% light transmittance is used. The light transmittance through the lampshade from the fluorescent lamp ranges from 80% to 90%. And 85% light transmittance is a balance between the participants' visual comfort and the intensity of the stimulus. What's more, the diameter of the hemispherical lampshade is 56mm which is just in the commonly used standard size. And the viewing distance is 70 cm in commonly studies. So the visual angle is 4.6 degrees which is a suitable visual angle for subjects. The stm32F103RB (STMicroelectronics company) is chosen as the microcontroller to produce multiple frequencies. By using the timer of STM32, the pulse width modulation (PWM) signal can be produced. Changing the duty ratio of the PWM signal changes the luminance of the LED. If the duty ratio of the PWM changes as DR (see formula (1)), the multi-frequency stimulation is possible. Besides, the timer is also used to guarantee the accuracy of the cycle. The time interval is 1 millisecond (ms).

$$DR=(450+(\sum_{i=1}^{n}A_{i}\times \sin(2\times \pi \times f_{i}\times t)))/900$$ (1)

where *f*_1_,*f*_2_,…,*f*_*n*_ are frequencies of the stimulation, *A*_1_,*A*_2_,…,*A*_*n*_ are amplitudes, *n* is the number of the multiple frequencies and *t* is time interval.

**Fig 1:**
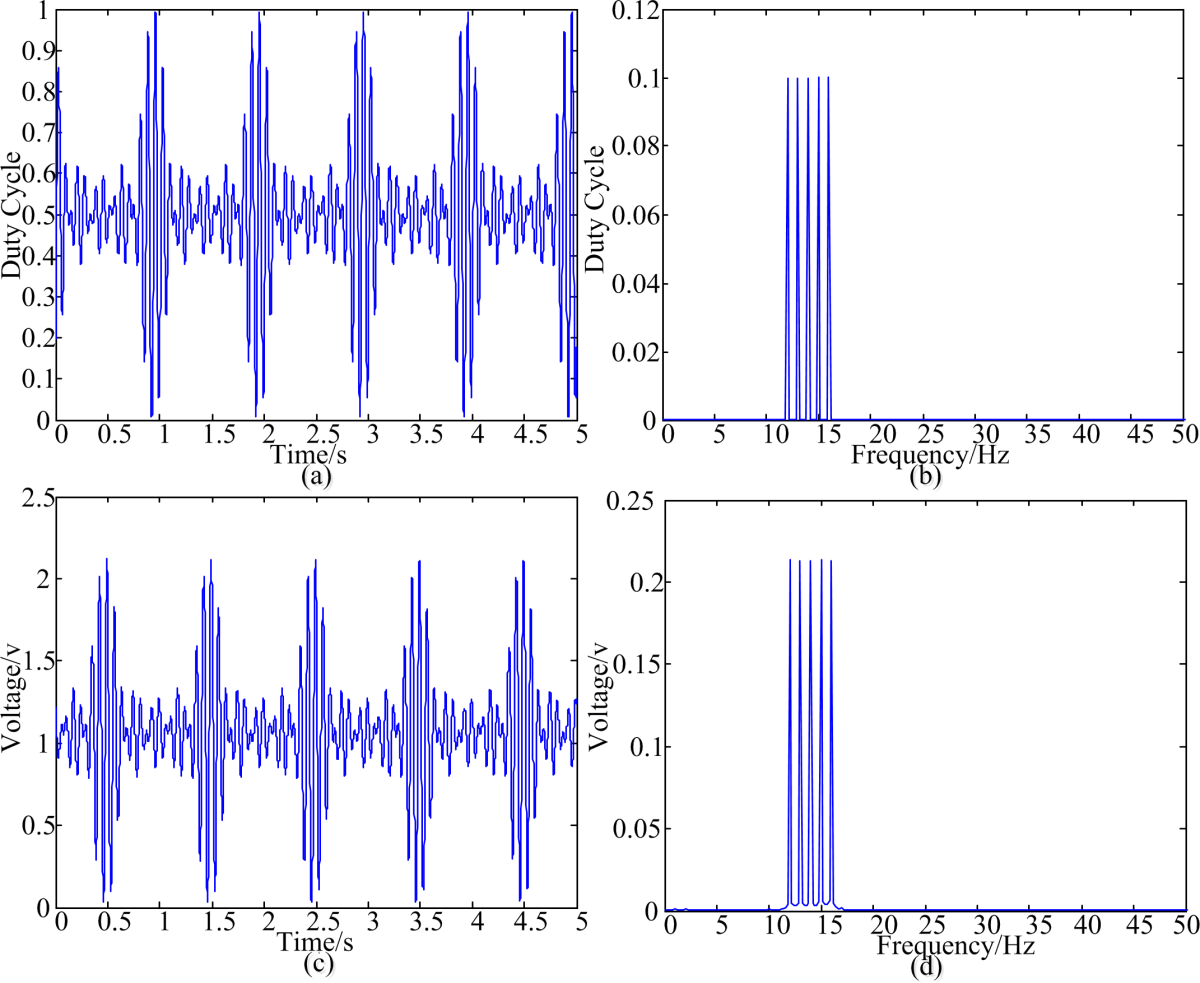
Luminance-based stimulation with 16Hz, 15Hz, 14Hz, 13Hz and 12Hz simultaneously. (a) duty ratio of the PWM (b) spectrum of the duty ratio (c) light intensity of LED flickering (d) spectrum of light intensity.

To guarantee the proposed method could produce the desired simultaneous flickering frequencies, the following test is performed prior to the experiments. Photistor (3DU5C) is chosen as the sensor to measure the change of light intensity of LED flickering. A resistor was connected to the photistor and a 3.3V power supply was used. The change in the resistance voltage was acquired by the data acquisition card NI9234 (National Instruments, USA). LED and photistor were placed face to face. The distance between them was 6 centimeter (cm). The simultaneous flickering frequencies were 16Hz, 15Hz, 14Hz, 13Hz and 12Hz. Fig 1 shows the result of the test. Fig 1A shows the simulation of duty ratio of PWM. Fig 1C shows the signal acquired by NI9234. Fig 1A and 1C are almost same. Their spectrums also have the same peak frequencies (Fig 1B and 1D respectively).

### Design of motion-based stimulation

**Fig 2:**
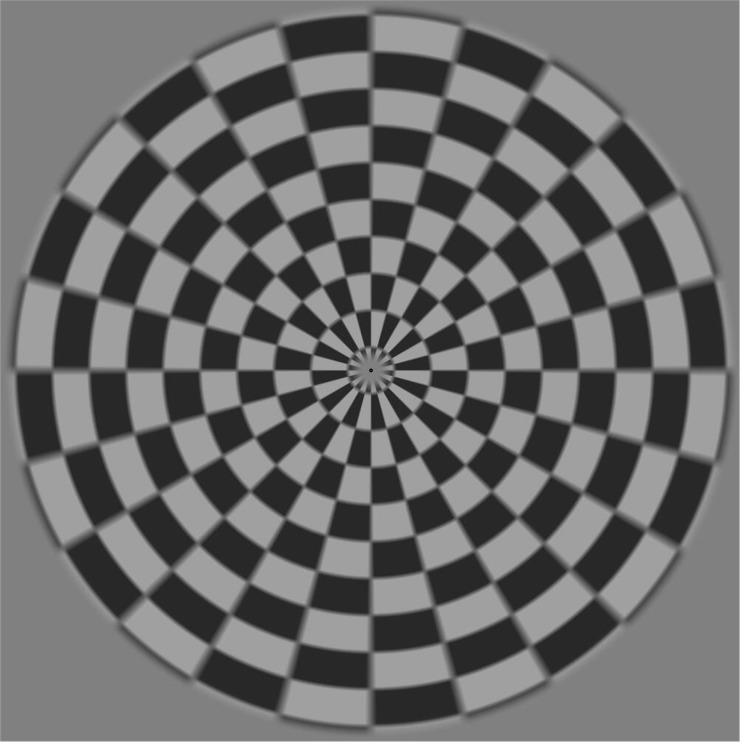
Motion-based stimulation.

The ring chessboard is chosen as the basic pattern of the proposed motion-based stimulation. The ring chessboard paradigm is a development of Newton’s rings paradigm [17] which can provide a comparable performance with low-adaptation characteristic and less visual discomfort for BCI applications. In the ring chessboard shown in Fig 2, each ring is divided into black and white lattices of equal numbers and sizes, so the area of the bright and dark areas in each ring are always equal. The brightness value of the central part of the stimulus unit is always set to the background brightness, and the area of the central part remains unchanged. As such, the luminance of the designed motion-based stimulation remains unchanged. The S1 File in the Supporting Information shows the main coding algorithms for producing the stimulation with dual frequencies (the style of the paradigm needs users to create it by themselves). The method to produce the chessboard paradigm proposed in this study is described below.

The stimulus program is developed under MATLAB using the Psychophysics Toolbox. Formula for generating pattern of ring checkerboard stimulating unit is as follows.

$$L=\{\begin{array}{l} C*\;\mathrm{sign}\;\{\cos[\pi \frac{r(x,y)}{W}+\varphi (t)\frac{A}{W}]*\;\cos[ang(x,y)*N_{C}]\}+L_{0},\;r>R_{\mathrm{inner}}\&r<R_{\mathrm{outer}}\\ \;L_{0}\;,\mathrm{others} \end{array}$$ (2)

where *φ*(t) is the phase value function in contraction expansion, *N*_*c*_ is the number of white and black squares contained in a single ring, *L*_0_ is background brightness, *W* is the width of the ring and *A* is the amplitude of the motion.

The motion reversal process of the motion-based stimulation is controlled by phase functions *φ*(t).

$$\varphi (t)=\frac{\pi }{2}+\sum_{i=1}^{n}\frac{\pi }{2\times i}\times \sin(2\times \pi \times f_{i}\times t-\frac{\pi }{2})$$ (3)

where *f*_1_,*f*_2_,…,*f*_*n*_ are frequencies of the stimulation. The ring chessboard will contract as the phase changes from 0 to pi and it will expand as the phase changes from pi to 0 (The specific forms of movement can refer to the S1 Video). Motion direction changes twice in one cycle. The motion inversion frequency is defined as the frequency of motion direction changes. And the motion inversion frequency is twice of motion frequency. The annular expansion and contraction of the ring chessboard forms visual stimuli with inter-modulation frequencies and the luminance remains unchanged.

### Experimental design

Ten healthy male subjects (ages 23–25) participated in the experiments. They were all volunteers from Xi'an Jiaotong University. EEG signals were recorded from six EEG electrodes (G.USBamp, G.tec Guger Technologies, Austria). Based on the international 10–20 system, the six EEG electrodes were placed at PO3, PO4, POz, O1, O2, Oz. The unilateral (left or right) earlobe was used as the recording reference and Fpz was used as ground. All electrodes' impedances were kept below 5 kOhm. The sampling frequency was 1200 Hz. Band-passed filter between 2 and 100 Hz and notch filter from 48Hz to 52Hz were used to remove artifacts and power line interface when acquiring EEG signals. The subjects were asked to sit on a comfortable chair in a quiet and ordinarily lit office room. All of the subjects had undergone BCIs before and had normal eyesight. Experiments were designed as described in Table 1.

**Table 1:** Experimental design.

	Multiple or dual inter-modulation frequencies	Ratio	Luminance or Motion	Frequency(Hz)	Duration(s)
E1	multiple	1:1	Luminance	16, 16+15, 16+15+14, 16+15+14+13, 16+15+14+13+12	5
	dual	1:3, 1:2, 1:1, 2:1, 3:1	Luminance	16+15	5
E2	dual	1:1	Luminance & Motion	5+8	10
E3	dual	1:1	Luminance	16+15, 16+14, 16+13, 16+17, 15+14, 15+13, 15+17, 14+13, 14+17, 13+17	1, 2, 3, 4, 5, 6
	dual	1:1	Motion	17.1+17.1, 17.1+15, 17.1+13.3, 17.1+12, 17.1+20, 15+15, 15+13.3, 15+12, 15+20, 13.3+13.3, 13.3+12, 13.3+20, 12+12, 12+20, 20+20	1, 2, 3, 4, 5, 6

#### E1: Offline experiment of brain response under luminance-based stimulation using inter-modulation frequencies

There were two tasks during this experiment: equal amplitude stimuli using multiple inter-modulation frequencies and unequal amplitude stimuli using fixed dual inter-modulation frequencies. The green LED with a hemispherical lampshade was placed 70 cm in front of the subject. The LED would flicker for 5 s and light out for 5 s automatically. All the subjects were required to stare at the flickering LED for 5 s per trial, 5 trials each inter-modulation frequency, with an interval of 5 s. Then the inter-modulation frequencies of the flickering LED would change as described below.

Task 1: equal amplitude stimuli using multiple inter-modulation frequencies. There were five different kinds of inter-modulation frequencies in this task including 16 Hz, 16+15 Hz, 16+15+14 Hz, 16+15+14+13 Hz and 16+15+14+13+12 Hz. The plus meant simultaneous multiple frequencies as formula (1) shows. The amplitudes of each frequencies were the same and the sum of the amplitudes was 450. As to 16+15+14+13+12 Hz, it means *f*_1_ = 16Hz, *f*_2_ = 15Hz, *f*_3_ = 14Hz, *f*_4_ = 13Hz, *f*_5_ = 12Hz and *A*_1_ = *A*_2_ = *A*_3_ = *A*_4_ = *A*_5_ = 90.

Task 2: unequal amplitude stimuli using fixed dual inter-modulation frequencies. There were also five different kinds of inter-modulation frequencies in this task. The frequencies were 16+15 Hz. But the amplitude of each frequency was different. The amplitudes were as follows: (*A*_1_ = 111, *A*_2_ = 333), (*A*_1_ = 150, *A*_2_ = 300), (*A*_1_ = 225, *A*_2_ = 225), (*A*_1_ = 300, *A*_2_ = 150), (*A*_1_ = 333, *A*_2_ = 111).

During all experiments, the EEG signals were recorded in the computer hard disk automatically.

#### E2: Offline experiment of comparisons between luminance-based and motion-based stimulation using dual inter-modulation frequencies

Luminance-based stimulation and motion-based stimulation described before were all used in this experiment. The inter-modulation frequencies were 5+8 Hz. All the subjects were required to stare at the two stimulations for 10 s per trial, with five trials for each stimulation, with an interval of 5 s respectively. As to luminance-based stimulation, the green LED with a hemispherical lampshade was placed 70 cm in front of the subject. And the LED would flicker for 10 s and then turned off for 5 s automatically. As for motion-based stimulation, it was presented in the middle of a LCD monitor. The diameter of the ring chessboard was 60 mm. The ring chessboard would for 10 s and then disappeared for 5 s automatically too. EEG signals were recorded in the computer hard disk automatically.

#### E3: Online experiment of luminance-based and motion-based stimulation using inter-modulation frequencies

In regard to luminance-based stimulation, there were ten targets with dual inter-modulation frequencies including 16+15 Hz, 16+14 Hz, 16+13 Hz, 16+17 Hz, 15+14 Hz, 15+13 Hz, 15+17 Hz, 14+13 Hz, 14+17 Hz, 13+17 Hz. As for motion-based stimulation, there were fifteen targets with frequencies including 17.1+17.1 Hz, 17.1+15 Hz, 17.1+13.3 Hz, 17.1+12 Hz, 17.1+20 Hz, 15+15 Hz, 15+13.3 Hz, 15+12 Hz, 15+20 Hz, 13.3+13.3 Hz, 13.3+12 Hz, 13.3+20 Hz, 12+12 Hz, 12+20 Hz, 20+20 Hz. The fifteen targets were presented on the monitor simultaneously. There were three rows and five targets in each row. The stimulation duration per trial was divided into six levels, from 1 s to 6 s with an interval of 1 s. Subjects were required to stare on the targets one by one. The luminance-based experiments were carried out for 5 runs and the motion-based experiments were carried out for 6 runs. And the identification results would be shown to subjects automatically during the interval.

### Data analysis

#### Canonical correlation analysis

Canonical Correlation Analysis (CCA) is widely used in SSVEP target recognition. It is carried out to calculate the correlations between reference signals and multi-channel EEG data [18]. The formula to calculate correlation coefficient *ρ* is as follows.

$$\rho =\max_{w_{x},w_{y}}\frac{E[w_{x}^{T}\;XY^{T}w_{y}]}{\sqrt{E[w_{x}^{T}\;XX^{T}\;w_{x}]E[w_{y}^{T}YY^{T}\;w_{y}]}}$$ (4)

where *X* is the EEG data, *Y* is the reference signals.

In this study, the reference signal *Y*_1_ in the luminance-based online experiment and the reference signal *Y*_2_ in the motion-based online experiment were chosen as follows:

$$Y_{1}=\left\{\begin{array}{c} \sin(2\times \pi \times f_{1}\times t)\\ \cos(2\times \pi \times f_{1}\times t)\\ \sin(2\times \pi \times f_{2}\times t)\\ \cos(2\times \pi \times f_{2}\times t) \end{array}\right\},Y_{2}=\left\{\begin{array}{c} \sin(\frac{f_{1}+f_{2}}{2})\\ \cos(\frac{f_{1}+f_{2}}{2}) \end{array}\right\}$$ (5)

where *Fs* is the sampling frequency and *f*_1_,*f*_2_ were the inter-modulation frequencies. Then the maximum of canonical coefficients was considered as the focused target. Besides, due to the latency delay in the visual system based on prior research [19], the date epochs began to be processed at 0.14 s.

#### CCA-based spatial filter

To enhance the signal-to-noise ratio, the CCA-based spatial filter was used to analyze the offline experimental data. When the correlations were calculated as in formula (4), the weight vectors *w*_*x*_ could be obtained at the same time. The multi channel EEG data can be converted into one-dimensional signals by multiplying the weight vectors *w*_*x*_ as follows.

$$\hat{X}=w_{x}^{T}X$$ (6)

#### Power spectrum density

The peak values at the stimulation frequencies in the power spectrum density (PSD) reflect the effect of inducing SSVEP by the designed stimulus. Hence the power spectrum density was chosen as the method to analyze the characteristics of the EEG response to multiple inter-modulation frequencies visual stimulus.

In this study, the Welch power spectrum density was used to estimate signals by dividing a datum with a length of *N* into *M* segments. The formula is as follows.

$$p(w)=\frac{1}{M}\sum_{i=1}^{M}\left(\frac{1}{lp_{0}}|\sum_{n=1}^{l}w(n)x_{i}(n)e^{-jwn}{|}^{2}\right)$$ (7)

#### Statistical analysis

Statistical analysis is conducted using repeated measures analysis of variance with Bonferroni posthoc analysis. Statistical significance is defined as *p* < 0.05.

## Results

### Offline experimental results of brain response under luminance-based stimulation using inter-modulation frequencies

**Fig 3:**
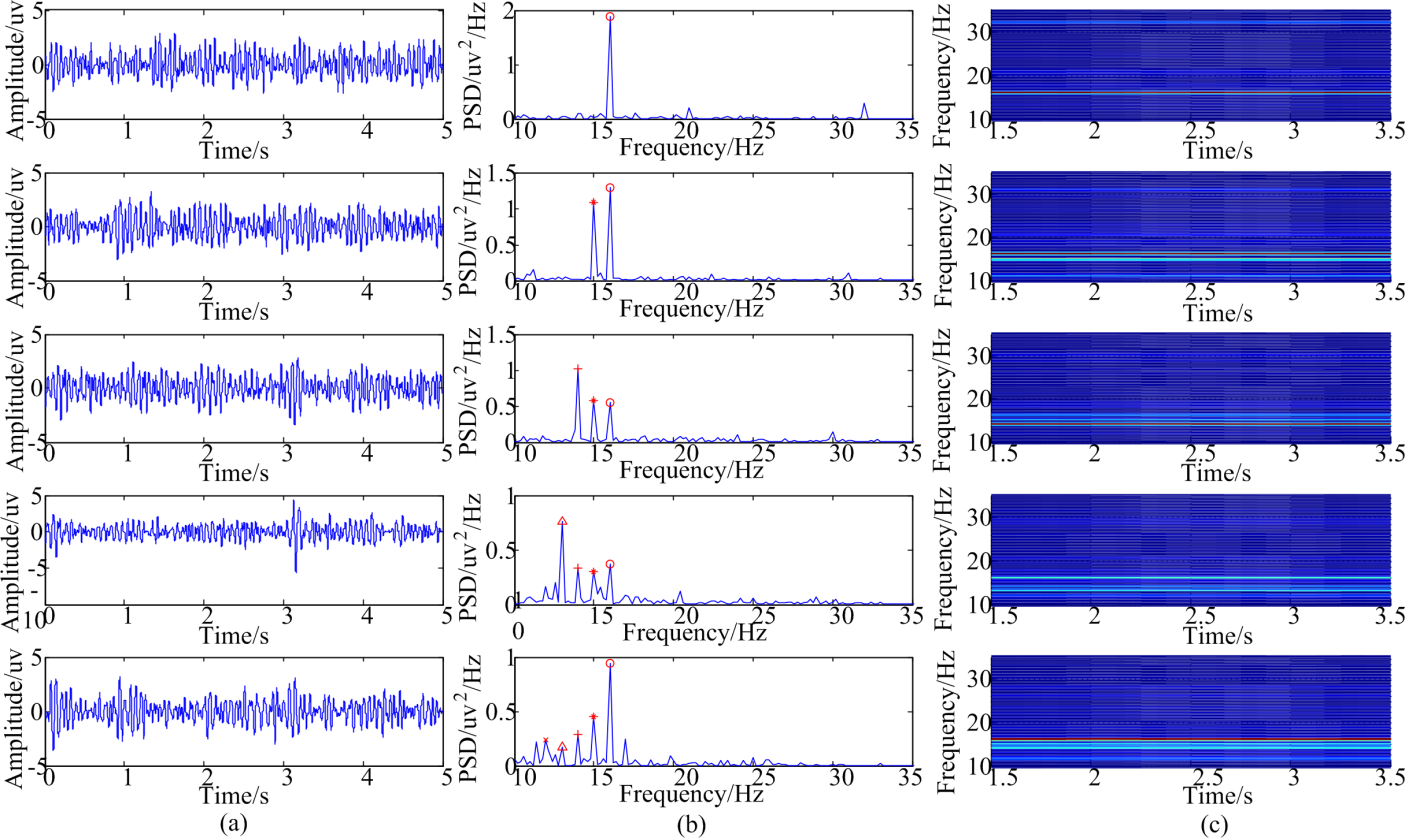
The time domain waveforms, power spectrum density and time-frequency maps acquired while a participant was staring at the equal amplitude stimulations. (a) The time domain waveforms. (b) Power spectrum density. (c) Time-frequency maps.

Fig 3 shows the time domain waveforms, PSD and time-frequency maps acquired while a participant was staring at the equal amplitude stimulation using multiple inter-modulation frequencies as described before. It was clearly observed from the PSD that the peaks were evoked at the frequencies of the stimuli. However, previous studies [20–22] showed that it would evoke the peaks at *m* × *f*_1_ + *n* × *f*_*h*_(*m*,*n* = 0,±1,±2…) where *f*_1_ and *f*_*h*_ were the dual inter-modulation frequencies. So as for the stimulus with 16+15+14 Hz, the peak at 14 Hz in the PSD may have been evoked by 14 Hz in the stimulus or in the combination frequency (14 = 2 × 15–16). So ANOVA on the peak values in the PSD was conducted to distinguish the difference. Table 2 shows the *P* values for all the subjects. Take subject 1 as an example, there was a significant difference on the peak values at 15 Hz in the PSD between the subject staring at the stimulation with 16 Hz and the stimulation with 16+15 Hz (*P* = 0.005<0.05). Similarly, there was significant difference on the peak values at 14 Hz in the PSD between the subject staring at the stimulation with 16+15 Hz and the stimulation with 16+15+14 Hz (*P* = 0.004<0.05). And there was a significant difference on the peak values at 13 Hz in the PSD between the subject staring at the stimulation with 16+15+14 Hz and the stimulation with 16+15+14+13 Hz (*P* = 0.029<0.05). Furthermore, there was a significant difference on the peak values at 12 Hz in the PSD between the subject staring at the stimulation with 16+15+14+13 Hz and the stimulation with 16+15+14+13+12 Hz (*P* = 0.04<0.05). The above mentioned results demonstrated that the stimulation would induce the corresponding frequencies in SSVEP when increasing frequencies in the inter-modulation frequencies stimulation and that the newly added induced frequencies in SSVEP were mainly caused by the added frequencies in the stimulation and not by the combination frequencies.

**Table 2:** ANOVA analyses on the peak values at the corresponding frequencies in the PSD while subjects stared at the stimulations with different kinds of frequencies.

	15 Hz	14 Hz	13 Hz	12 Hz
S1	*P* = 0.005	*P* = 0.004	*P* = 0.029	*P* = 0.04
S2	*P* = 0.000	*P* = 0.01	*P* = 0.225	*P* = 0.031
S3	*P* = 0.019	*P* = 0.015	*P* = 0.019	*P* = 0.023
S4	*P* = 0.01	*P* = 0.021	*P* = 0.006	*P* = 0.012
S5	*P* = 0.006	*P* = 0.008	*P* = 0.002	*P* = 0.041
S6	*P* = 0.005	*P* = 0.046	*P* = 0.003	*P* = 0.012
S7	*P = 0*.*000*	*P = 0*.*003*	*P = 0*.*026*	*P = 0*.*001*
S8	*P = 0*.*000*	*P = 0*.*021*	*P = 0*.*002*	*P = 0*.*012*
S9	*P = 0*.*000*	*P = 0*.*001*	*P = 0*.*018*	*P = 0*.*012*
S10	*P = 0*.*000*	*P = 0*.*003*	*P = 0*.*003*	*P = 0*.*000*

**Fig 4:**
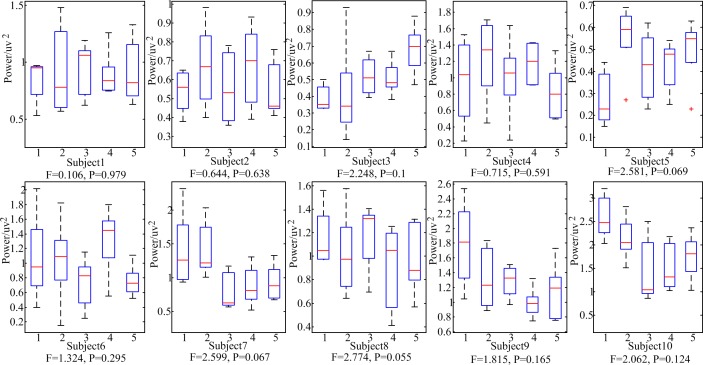
The variance maps about the sum of the peak values at the stimulus frequencies in the PSD.

Even though the amplitudes of the stimulus at each frequency were equal, the peak values at the corresponding frequencies in PSD evoked by the stimulus were not the same. There was an interesting discovery in this result. The sum of PSD at the stimulus frequencies was calculated. Fig 4 shows the variance maps of the sum of PSD at the stimulus frequencies. The '1' in x axis indicates the sum of peak values at 16 Hz in PSD. The '2' in x axis indicates the sum of peak values at 16 Hz and 15 Hz in PSD. The '3' in x axis indicates the sum of peak values at 16 Hz, 15 Hz and 14 Hz in PSD. The '4' in x axis indicates the sum of peak values at 16 Hz, 15 Hz, 14 Hz and 13 Hz in PSD. The '5' in x axis indicates the sum of peak values at 16 Hz, 15 Hz, 14 Hz, 13 Hz and 12 Hz in PSD. The results showed that there were no significant difference between the different sums of PSD for each subject (*P*>0.05). That meant the sum of the peak values at the multiple inter-modulation frequencies in the PSD demonstrated no significant difference.

**Fig 5:**
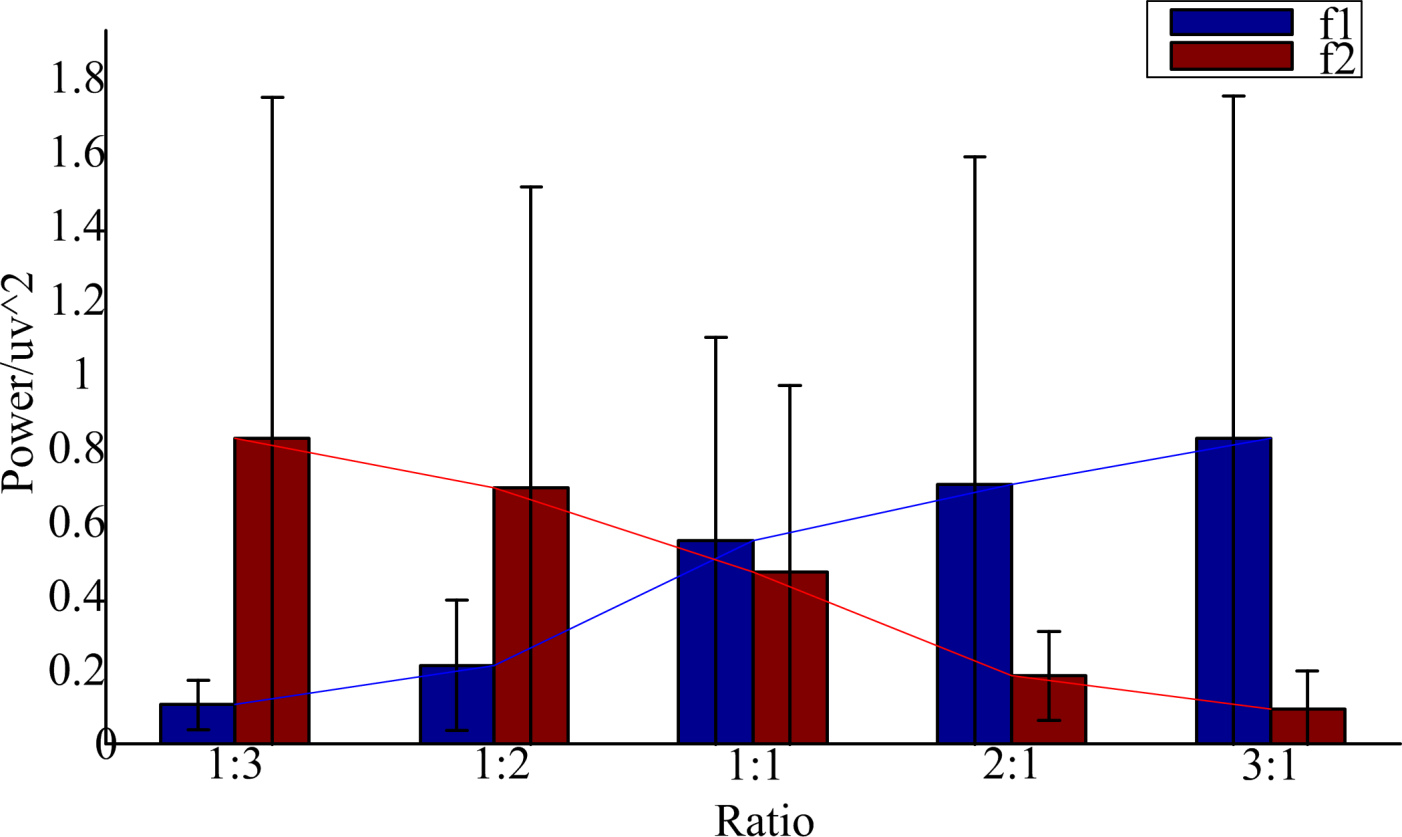
The sum of peak values at 15 Hz in the PSD among all the participants and peak values at 16 Hz in the PSD among all the participants.

**Fig 6:**
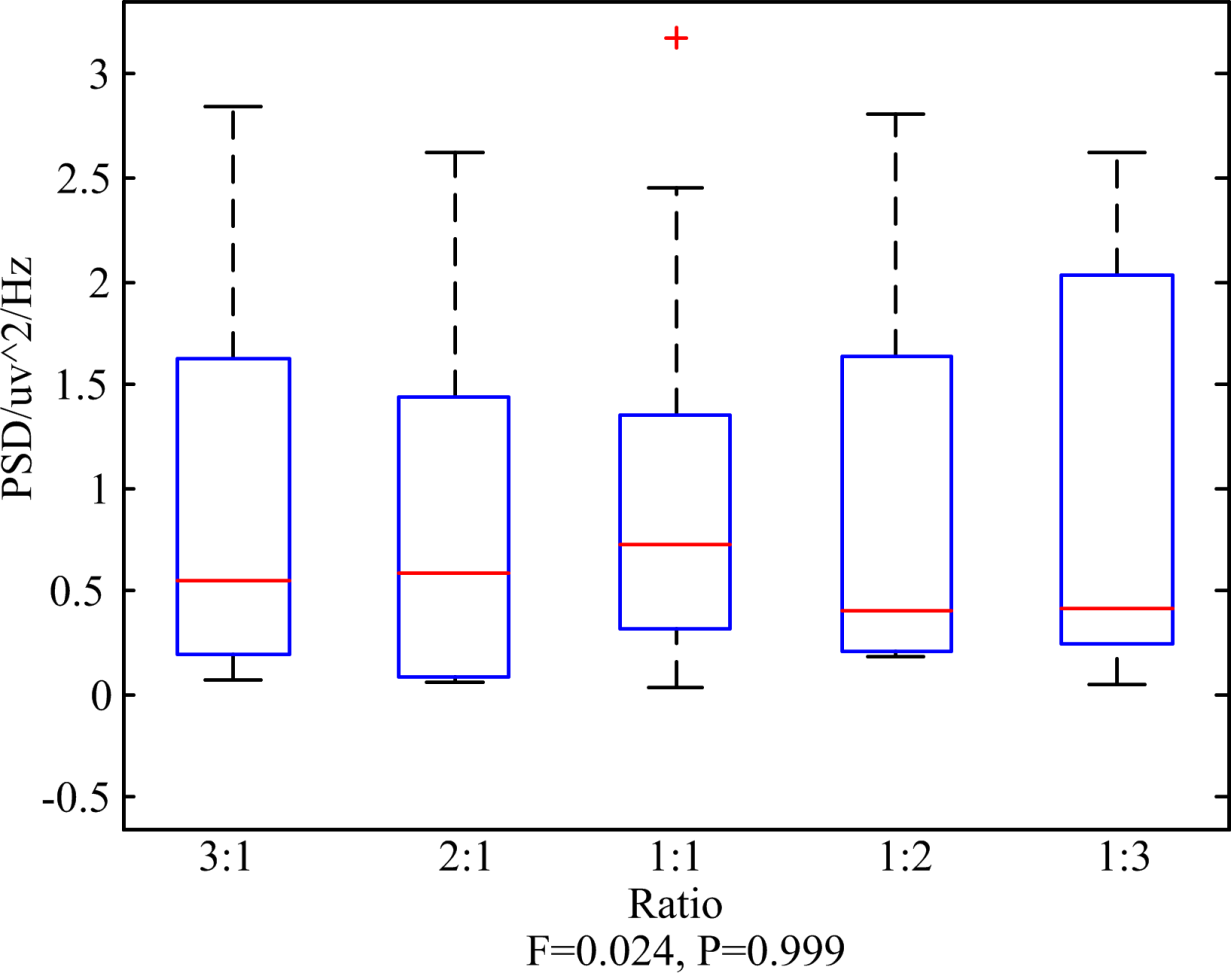
The variance maps of the sum of peak values at 15 Hz and 16 Hz in the PSD among all the participants.

Fig 5 shows the mean of the peak values at 15 Hz in the PSD among all the participants and the mean of the peak values at 16 Hz in the PSD among all the participants while they were staring at the unequal amplitude stimulation using dual-modulation frequencies as described before. As the increase of one amplitude of the stimulation using dual inter-modulation frequencies, the peak values at corresponding frequency in the PSD increased. However the sum of the peak values at 15 Hz and 16 Hz in the PSD had no significant difference (*P* = 0.999>0.05) as shown in Fig 6. As such, it was concluded that the sum of the peak values at the two frequencies in the PSD had no significant difference as to unequal amplitude stimuli using fixed dual inter-modulation frequencies. That's to say the brain response had a positive correlation with the light intensity of the stimulation using inter-modulation frequencies.

### Offline experimental results of brain response under luminance-based stimulation and motion-based stimulation using inter-modulation frequencies

**Fig 7:**
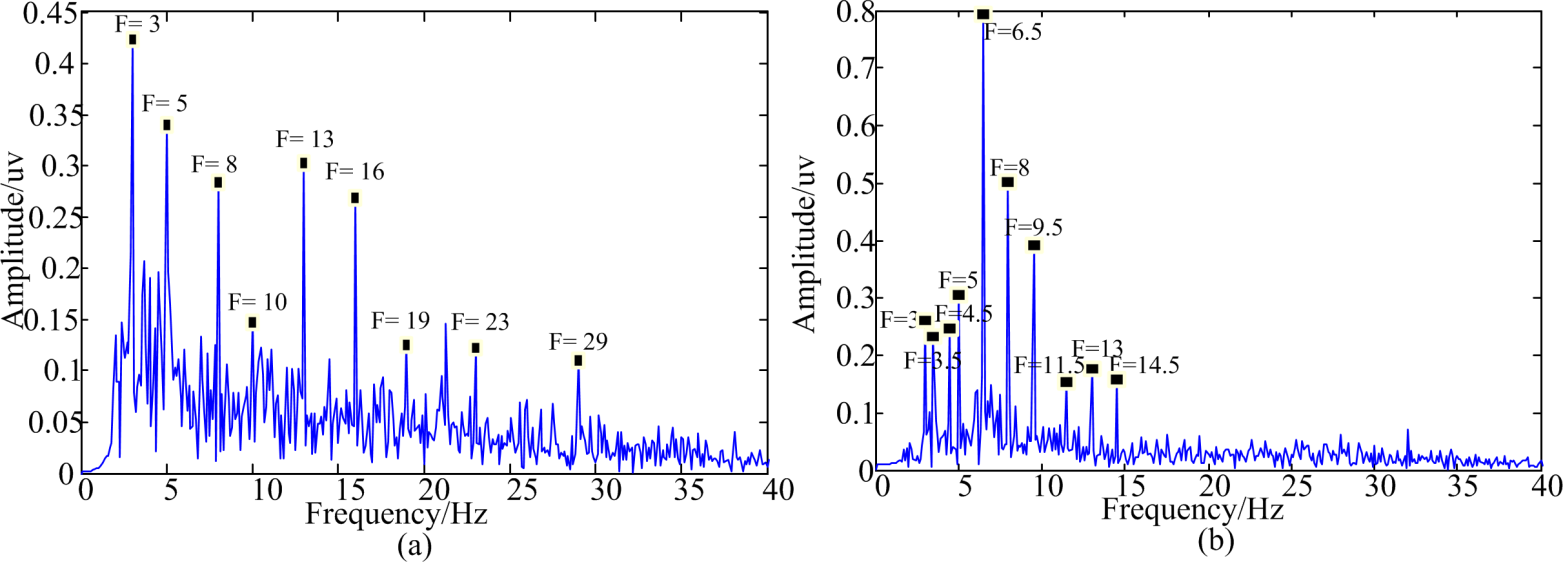
Frequency spectra of EEG data at the Oz site dealt with using time domain averaging on luminance-based and motion-based stimulation using dual inter-modulation frequencies. (a) Frequency spectra of EEG data at the Oz site with luminance-based stimulation using dual inter-modulation frequencies. (b) Frequency spectra of EEG data at the Oz site with motion-based stimulation using dual inter-modulation frequencies.

The Oz site experiences the main neuronal activities in the visual area because it is closest to the striate cortex [23]. Fig 7 shows the frequency spectrums of EEG data at the Oz site while one participant was staring at the luminance-based stimulation and motion-based stimulation respectively. The EEG data were dealt with using time domain averaging first. Several combination frequencies can be clearly observed in both spectrum. As for luminance-based stimulus, the peaks in the spectrum occurred at *f*_1_,*f*_2_,2 × *f*_1_,2 × *f*_2_,*f*_2_±*f*_1_,3 × *f*_2_ ± *f*_1_,3 × *f*_1_ + *f*_2_ where *f*_1_ = 5*Hz*, *f*_2_ = 8*Hz*. As for motion-based stimulus, the peaks in the spectrum occurred at $f_{1},f_{2},\frac{\left(f_{2}+f_{1}\right)}{2},f_{2}\pm f_{1},\frac{\left(3\times f_{2}\pm f_{1}\right)}{2},\frac{\left(3\times f_{1}\pm f_{2}\right)}{2}$ where *f*_1_ = 5*Hz*, *f*_2_ = 8*Hz*. So both the luminance-based and motion-based stimulation using dual inter-modulation frequencies could induce SSVEP at combination frequencies. But there were some differences between them. The induced frequencies by motion-based stimulation were combined by the motion frequencies $\left(\frac{f_{1}}{2},\frac{f_{2}}{2}\right)$, not the motion inversion frequencies. And the peaks occurred at the motion inversion frequencies, not the motion frequencies.

### Experimental results of online recognition accuracy

**Fig 8:**
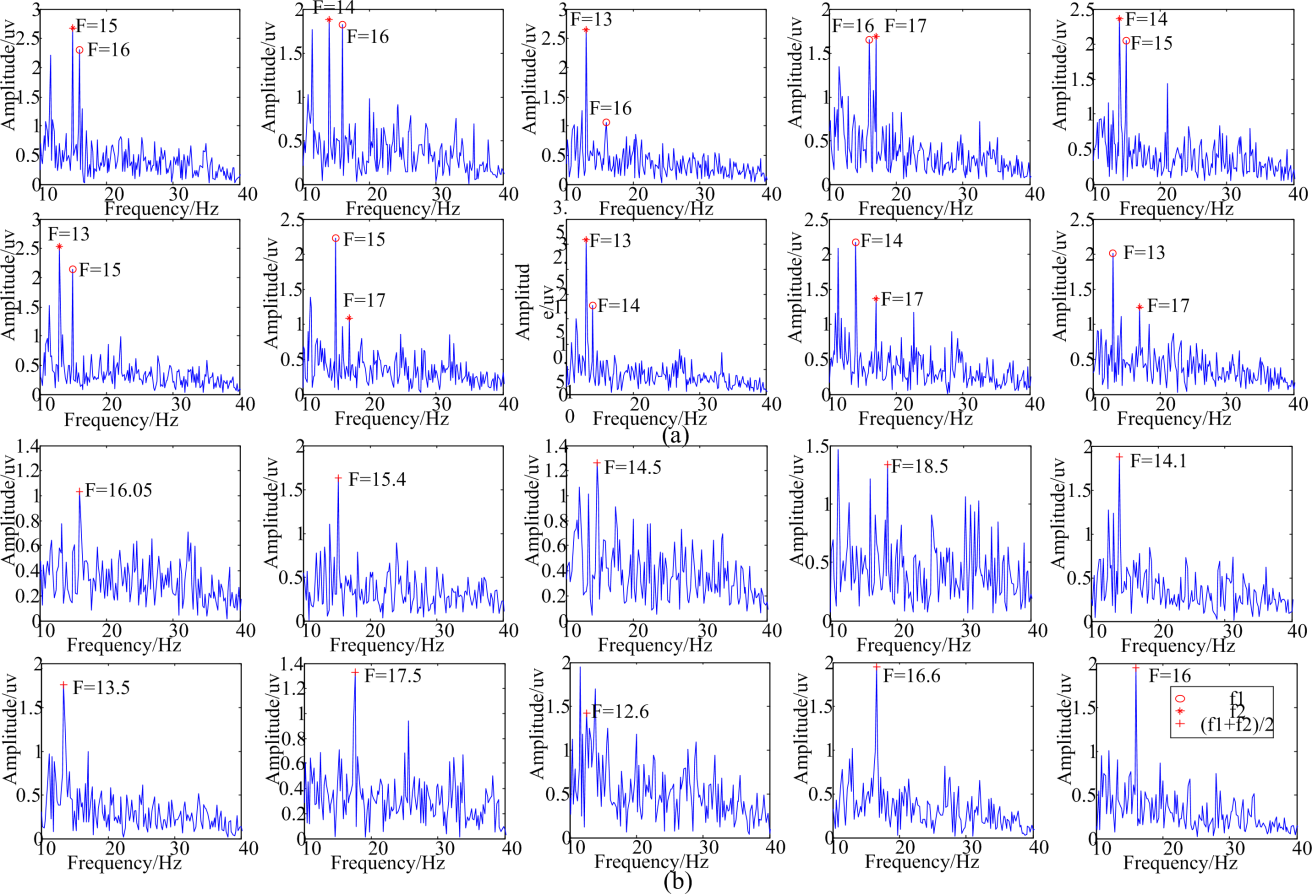
Frequency spectrum of EEG data for a single trial at the Oz site with luminance-based and motion-based stimulation using dual inter-modulation frequencies. (a) Frequency spectra of EEG data for a single trial at the Oz site with luminance-based stimulation using dual inter-modulation frequencies. (b) Frequency spectra of EEG data for a single trial at the Oz site with motion-based stimulation using dual inter-modulation frequencies.

Fig 8 shows the frequency spectrum of EEG data of a single trial at Oz site with luminance-based and motion-based stimulation using dual inter-modulation frequencies. When the dual frequencies were 16+15 Hz, 16+14 Hz, 16+13 Hz, 16+17 Hz, 15+14 Hz, 15+13 Hz, 15+17 Hz, 14+13 Hz, 14+17 Hz, and 13+17 Hz in proper order, in regard to luminance-based stimulation, the main induced frequencies were (16 Hz 15 Hz), (16 Hz 14 Hz), (16 Hz 13 Hz), (16 Hz 17 Hz), (15 Hz 14 Hz), (15 Hz 13 Hz), (15 Hz 17 Hz), (14 Hz 13 Hz), (14 Hz 17 Hz), and (13 Hz 17 Hz) successively as shown in Fig 8(A). When the dual frequencies were 17.1+15 Hz, 17.1+13.3 Hz, 17.1+12 Hz, 17.1+20 Hz, 15+13.3 Hz, 15+12 Hz, 15+20 Hz, 13.3+12 Hz, 13.3+20 Hz, and 12+20 Hz in proper order, as to motion-based stimulation, the main induced frequencies were 16.05 Hz, 15.4 Hz, 14.5 Hz, 18.5 Hz, 14.1 Hz, 13.5 Hz, 17.5 Hz, 12.6 Hz, 16.6 Hz, 16 Hz successively as shown in Fig 8(B).

In total, as to luminance-based stimulation, the main peaks in the spectrum occurred at *f*_1_ and *f*_2_. The amplitudes at the combination frequencies in the luminance-based stimulation were not outstanding. So *Y*_1_ in forum (5) was chosen as the reference signals to perform CCA. As to motion-based stimulation, the main peaks in the spectrum occurred at $\frac{f_{1}+f_{2}}{2}$. So *Y*_2_ in forum (5) was chosen as the reference signals to perform CCA.

CCA was used to calculate the recognition accuracy of luminance-based stimulation and motion-based stimulation for each subjects. The recognition accuracies of luminance-based stimulation and motion-based stimulation of all the subjects according to stimulation duration are shown in Tables 3 and 4 respectively. Noticeably, the recognition accuracy of S10 reached 98% with only 2 s of stimulation. Regarding the average accuracies, the luminance-based stimulation had higher recognition accuracy than the motion-based stimulation did when the duration was lower than 4 s. And both the recognition accuracies of the two stimulations were decrease sharply as the duration decreased. When the stimulation duration exceeded 4 s, the change of recognition accuracy tended to be stable and the motion-based stimulation had higher recognition accuracy than the luminance-based stimulation did.

**Table 3:** Online classification accuracy of luminance-based stimulation according to stimulation duration.

	6 s	5 s	4 s	3 s	2 s	1 s
S1	0.82	0.76	0.78	0.74	0.72	0.56
S2	0.78	0.78	0.76	0.72	0.58	0.46
S3	0.92	0.94	0.94	0.88	0.8	0.6
S4	0.98	0.96	0.9	0.74	0.56	0.26
S5	0.96	0.94	0.94	0.9	0.76	0.48
S6	1	1	1	1	0.92	0.74
S7	1	1	1	0.95	0.85	0.45
S8	1	0.9667	1	0.9333	0.8333	0.4667
S9	0.98	0.96	0.88	0.78	0.48	0.26
S10	1	1	1	1	0.98	0.68
Mean ± std	0.9440 ± 0.0804	0.9307 ± 0.0878	0.9200 ± 0.0904	0.8643 ± 0.1102	0.7483 ± 0.1633	0.4957 ± 0.1578

**Table 4:** Online classification accuracy with motion-based stimulation according to stimulation duration.

	6 s	5 s	4 s	3 s	2 s	1 s
S1	0.9333	0.8889	0.9	0.8333	0.7445	0.3667
S2	0.9778	0.9556	0.9222	0.8445	0.7112	0.3777
S3	0.9222	0.8889	0.8	0.7555	0.6	0.4111
S4	0.9889	0.9778	0.9889	0.9444	0.8667	0.5332
S5	0.8667	0.8333	0.8445	0.7778	0.666	0.3888
S6	0.9444	0.9333	0.8444	0.7333	0.5889	0.1889
S7	1	0.9889	0.9444	0.8222	0.7333	0.4111
S8	0.9889	0.9778	0.9667	0.9333	0.7778	0.4889
S9	1	1	0.9667	0.8	0.7	0.2667
S10	0.9833	0.9833	0.9833	0.8834	0.7667	0.4167
Mean±std	0.9605±0.0432	0.9428±0.0553	0.9161±0.0665	0.8328±0.0710	0.7155±0.0834	0.3850±0.0989

**Fig 9:**
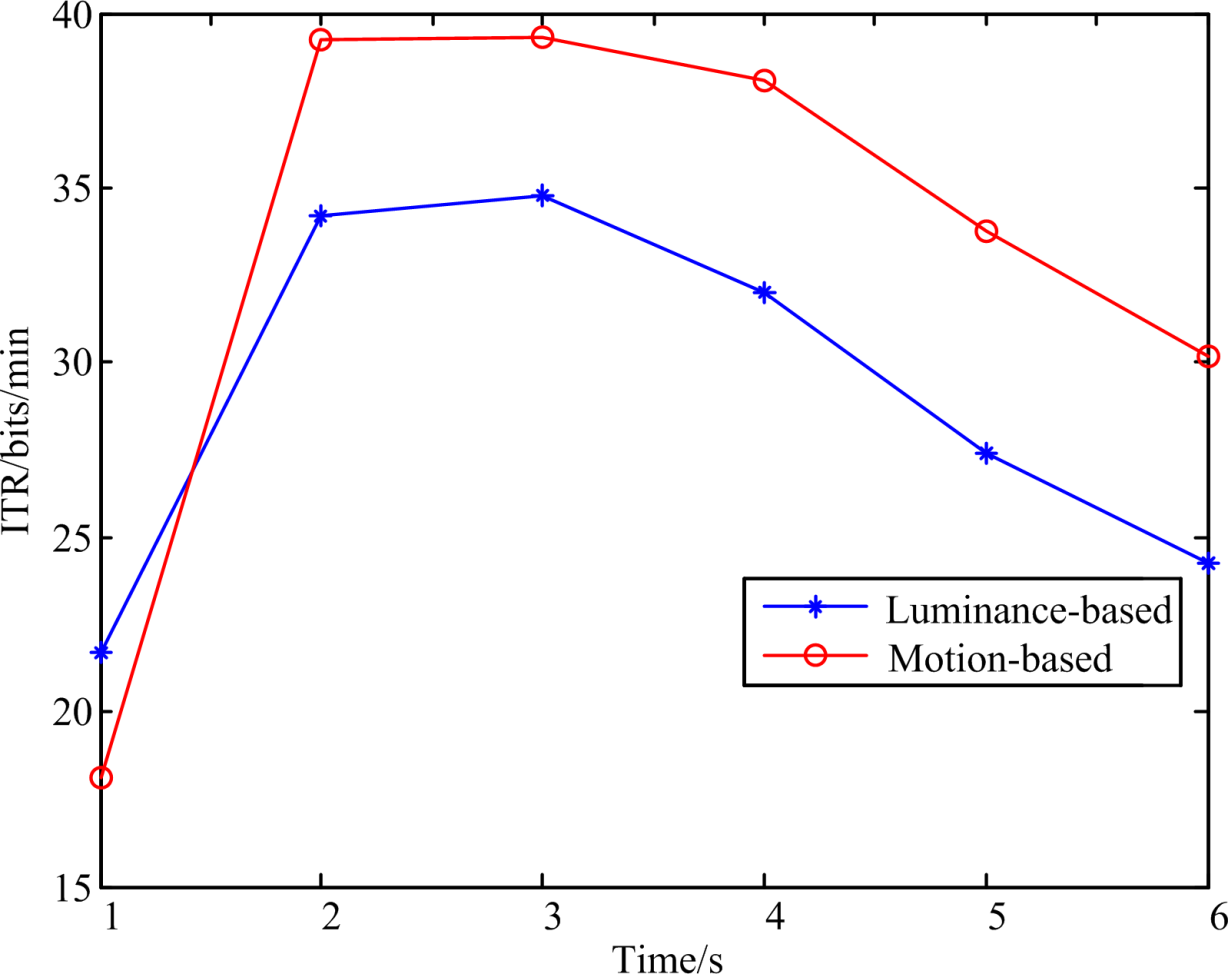
Mean of ITR of all the subjects with different stimulation duration.

Fig 9 shows the mean information transfer rate (ITR) of subjects with different stimulation duration. The ITR reached the highest value at 3 s for 34.7836 bits/min and 39.2856 bits/min with luminance-based and motion-based stimulation respectively. In total, the ITRs of the motion-based stimulation were higher than that of the luminance-based stimulation for the different durations except 1 s.

## Discussion

In this study, luminance-based and motion-based stimulations using inter-modulation frequencies were proposed and these two methods were introduced as new tools for BCI systems to extend related research. Characteristics of EEG response to luminance-based stimulation and motion-based stimulation using multiple inter-modulation frequencies were illustrated. Both offline analysis and online testing demonstrated the feasibility of the proposed methods to encode more targets with limited frequencies.

The inter-modulation frequencies were first reported by Ratliff's group [24]. Regan [20] described the nonlinearity of the EEG response to stimulus using dual inter-modulation frequencies. Several follow-up studies reported similar results. T M Srihari Mukesh [25] proposed a novel dual frequency stimulation method to increase the number of selections in BCI. Only three frequencies generated six selections in their study. And the peaks in the spectrum mainly occurred at *F*_1_,*F*_2_,*F*_1_ + *F*_2_,2 × *F*_2_,*F*_1_ + 2 × *F*_2_, 2 × *F*_1_+ 2 × *F*_2_,3 × *F*_2_,*F*_1_ + 3 × *F*_2_,2 × *F*_1_ + 3 × *F*_2_. Shyu et al. [11] obtained a dual-frequency stimulation with two flickering LEDs. The peaks mainly occurred at *F*_1_,*F*_2_,2 × *F*_2_–*F*_1_,2 × *F*_1_–*F*_2_. Min Hye Chang, etc [26] proposed an amplitude-modulated visual stimulation which combined the advantage of both low and high frequencies with dual frequencies. And the peaks occurred at 2 × *f*_*c*_,2 × *f*_*m*_,*f*_*c*_ ± *f*_*m*_,*f*_*c*_ ± 3 × *f*_*m*_,2 × *f*_*c*_ ± 4 × *f*_*m*_. All the studies motioned above were dual frequencies stimulus and all the induced frequencies reflected the nonlinearity of the brain. This study extended dual frequencies to multiple frequencies. The results demonstrated that simultaneous modulation of stimulus luminance with multiple frequencies can induce the corresponding frequencies. And at least five stimulus frequencies could induced simultaneously. What's more, the combination frequencies were not significant. That is to say the nonlinearity of the brain response to our proposed stimulus using multiple inter-modulation frequencies from 12 Hz to 16 Hz was not obvious. What's more, our results showed the brain had a certain positive correlation with luminance even using inter-modulation frequencies. With the increase of one amplitude of the stimulation using inter-modulation frequencies, the peak values at corresponding frequencies in the PSD increased. When the sum of amplitudes at the stimulus frequencies of the stimulus was kept the same, the sum of peak values at corresponding frequencies in the PSD of EEG demonstrated no significant difference.

On the other hand, in Xiaogang Chen's research [22, 27], not only simultaneous modulation of stimulus luminance, but also of stimulus color were considered. *F*_*i*_ ± *f*_*i*_,2*F*_*i*_ ± *f*_*i*_ were the main inter-modulation frequencies in their study. It was known that luminance and color are all reflected in the P-pathway in primary visual cortex. However, the motion-based stimulation was reflected in the M-pathway. So in order to get a comprehensive understanding of the primary visual cortex response to visual stimulus, motion-based stimulation with equal luminance using inter-modulation frequencies was proposed in this study. The results in this study showed that the brain response to the proposed motion-based stimulation and luminance-based stimulation using inter-modulation frequencies had certain similarities in low frequency bands. Both stimulation methods of them induced combination frequencies. And the results showed the nonlinearity of the brain. But the induced frequencies by motion-based stimulation were combined by the motion frequencies, not the motion inversion frequencies. And there were no peaks that occurred at the motion frequencies. Besides it was still unknown how these frequencies were produced. Zhenghua Wu [28] proposed one theory that different SSVEP neural networks exist whose strongest response are located in different frequency bands by comparing the SSVEP power under a single-eye stimulus and a simultaneous, dual-eye stimulus. A hypothesis was given in this study. The retina received the visual stimulus signal *IS*. Due to the brain resonances [29], we supposed that the input signal *IS* was changed into *NS* when the visual stimulus was transferred to the primary visual cortex. *NS* contained the abundant inter-modulation frequencies *f*_1_ ± *f*_2_ (*i* = 2), 2 × *f*_1_ ± *f*_2_,2 × *f*_2_ ± *f*_1_ (*i* = 3), 3 × *f*_1_ ± *f*_2_,3 × *f*_2_ ± *f*_1_ (*i* = 4) and so on.

$$IS=A_{1}\times \sin(2\times \pi \times f_{1}\times t)+A_{2}\times \sin(2\times \pi \times f_{2}\times t)$$ (8)

$$NS=\sum_{i=1}^{n}B_{i}\times {\left(A_{1}\times \sin(2\times \pi \times f_{1}\times t)+A_{2}\times \sin(2\times \pi \times f_{2}\times t)\right)}^{i}$$ (9)

Due to different sensitivities of the brain to different frequencies, some combination frequencies did not occur. And the amplitudes of the combination frequencies were different. So induced combination frequencies reported by other studies were different.

Besides, simultaneous modulation of stimulus was used to increase the number of targets using limited frequencies. Actually, n frequencies could totally generate $C_{n}^{1}+C_{n}^{2}+C_{n}^{3}+\ldots +C_{n}^{n}$ selections in our study. And our stimulation only contained one flickering LED or one target with multiple frequencies which did not cause the 'attention-shift' problem noted in previous studies [10, 11, 30]. What's more, Hakvoort [31] mentioned that more than 16% of the selections were misclassified because of the multiple harmonics using conventional CCA. In this study, *Y*_1_ and *Y*_2_ in formula (5) were chosen as the reference signals for the luminance-based and motion-based stimulations respectively. And online test showed the average ITRs reached 34.7836 bits/min and 39.2856 bits/min respectively. This meant that reference signals using CCA should be chosen based on the stimulus. In regard to ITR, the motion-based stimulation was better than the luminance-based stimulation. But the motion-based stimulation was difficult to extend to multiple inter-modulation frequencies and only had a regular rule with dual inter-modulation frequencies. This was not the case with luminance-based stimulation. For the further research, the classification algorithms, stimulus frequencies, and induced frequencies still need to be optimized to increase the ITR and recognition accuracy.

## Conclusion

The present study presented a thorough investigation on stimulation for SSVEP using inter-modulation frequencies. Importantly, luminance-based stimulation using one to five inter-modulation frequencies could induce the corresponding stimulus frequencies in the primary visual cortex. And the response maintained a certain positive correlation with luminance. Additionally, the brain response to motion-based stimulation with equal luminance had similar characteristics with that of luminance-based stimulation. And a hypothesis was presented to explain the reason for the occurrence of combination frequencies. Furthermore, the ITRs in the online test indicated that both of the proposed stimulations were feasible for multi-class SSVEP-BCI systems.

## Supporting information

S1 File The main coding algorithms for producing the stimulation with dual frequencies. (M)

S2 File The peak values of PSD at corresponding stimulus frequencies in E1 task1. (SAV)

S3 File The sum of the peak values of PSD at corresponding stimulus frequencies in E1 task1. (SAV)

S4 File The sum of peak values of PSD at 15 Hz and 16 Hz in E1 task2. (SAV)

S5 File The frequency spectrum of EEG data in E2. (XLSX)

S6 File The frequency spectrum of EEG data in E3. (XLSX)

S7 File The mean of ITR of all the subjects with different stimulation duration. (XLSX)

S1 Video The chessboard stimulation using dual inter-modulation frequencies. (MP4)
